# Dr. John Wing Brown

**Published:** 1914-10

**Authors:** 


Dr. John Wing Brown, Michigan, 1873, died at his home in Mottville, Onondaga Co., N. Y., July 9, aged 62.

